# Biomarker suPAR seems a good prognostic factor for community-acquired pneumonia but less prominent for septic shock

**DOI:** 10.1186/s13054-019-2694-0

**Published:** 2019-12-11

**Authors:** Patrick M. Honore, Aude Mugisha, Leonel Barreto Gutierrez, Sebastien Redant, Keitiane Kaefer, Andrea Gallerani, David De Bels

**Affiliations:** ICU Department, Centre Hospitalier Universitaire, Place Van Gehuchtenplein,4, 1020 Brussels, Belgium

We read with interest the article by Luo et al. [1]. The urokinase-type plasminogen activator system consists of a protease, a receptor urokinase-type plasminogen activator receptor (uPAR), and inhibitors [2]. Depending on the degree of glycosylation and proteolytic cleavage, soluble urokinase-type plasminogen activator receptor (suPAR) is a circulating protein ranging mostly between 20 and 35 kDa [2]. Luo et al. show that suPAR exhibits high accuracy for both diagnosis and prognosis of severe community-acquired pneumonia (CAP) [1]. We would like to make some comments. While the area under the curve (AUC) for suPAR in order to accurately differentiate severe CAP (SCAP) from CAP is extremely good (AUC of 0.835 (*p* < 0.001) [1], it was reported poor regarding its ability to differentiate severity of sepsis shock (AUC of 0.62) [3]. An explanation could be that nearly half of critically ill patients especially with septic shock have or develop acute kidney injury (AKI) and 20–25% needs renal replacement therapy (RRT) within the first week of their stay [4]. In the study of Luo, only 22 out of the 103 SCPA patients were in septic shock, so the rate of AKI and CRRT was much lower in Luo’s cohort when compared to a full septic shock cohort [4]. Continuous RRT (CRRT) is performed using membranes that have a cut value of 35–40 kDa, and therefore, some quantity of suPAR will be eliminated [5]. New highly adsorptive membranes (HAM) that can adsorb many molecules with a molecular weight above 35 kDa will even increase this removal [5]. This can mislead patient prognostication by artificially decreasing suPAR, but no studies have challenged this issue. Such studies should be done as there is already a long list of biomarkers in sepsis that are lacking reliability during CRRT [5]. To date, no single sepsis biomarker can be reliable during CRRT. While suPAR might be a good marker of severity in SCAP, this might not be the case for septic shock. A hypothesis that could explain this discrepancy could be the rate of CRRT use in this group of patients that is much higher as compared to SCAP in the Luo’s study. As a consequence of the high CRRT rate, suPAR levels are no longer reliable in septic patients [1].

## Data Availability

Not applicable.

## Abbreviations

uPAR: Urokinase-type plasminogen activator receptor
suPAR: Soluble urokinase-type plasminogen activator receptor
CAP: Community acquired pneumonia
SCAP: Severe community-acquired pneumonia
AUC: Area under the curve
AKI: Acute kidney injury
RRT: Renal replacement therapy
CRRT: Continuous renal replacement therapy
HAM: Highly adsorptive membranes
